# Agreement Between Manual and Digital Mesiodistal Tooth Measurements on Digitized Plaster Models: A Tooth-Level Analysis

**DOI:** 10.3390/jcm15155911

**Published:** 2026-07-29

**Authors:** Helena Brawańska, Wiktoria Kukla, Viktoryia Serha, Marcin Gruca, Agata Gawrychowska-Janicka, Agnieszka Machorowska-Pieniążek

**Affiliations:** Department of Orthodontics, Faculty of Medical Sciences in Zabrze, Medical University of Silesia, 40-055 Katowice, Poland; wiktoriakukla.a@gmail.com (W.K.); s89317@365.sum.edu.pl (V.S.);

**Keywords:** digital models, intraoral scanner, orthodontic diagnosis, mesiodistal tooth width, measurement agreement, Bland–Altman analysis

## Abstract

**Background:** Reliable mesiodistal tooth-width measurements are essential for orthodontic diagnosis and treatment planning. Digital models obtained by scanning plaster casts may facilitate orthodontic workflows; however, the extent of agreement between manual caliper measurements and digital measurements on digitized plaster models remains clinically relevant, particularly at the individual-tooth level. **Objective:** To evaluate the agreement, repeatability, and inter-method differences between manual and digital mesiodistal tooth-width measurements from the canine to the first molar on digitized plaster models. **Methods:** Plaster models from 30 orthodontic patients were analyzed. The models were digitized using a 3Shape TRIOS 4 intraoral scanner. Mesiodistal widths of teeth 13–16, 23–26, 33–36, and 43–46 were measured using digital calipers on plaster models and using 3Shape Unite platform version 1.7.33.2 (3Shape A/S, Copenhagen, Denmark) on the corresponding digital models. Manual and digital measurements were each performed twice at 24 h intervals. For both methods, the mean of the two repeated measurements was used for inter-method comparison. Manual and digital intra-observer repeatability was assessed using the intraclass correlation coefficient (ICC). Inter-method differences (ΔMD = mean manual − digital) were analyzed using Bland–Altman analysis. Differences exceeding 0.3 mm were considered to exceed the predefined threshold of potential clinical relevance. **Results:** A total of 480 teeth were analyzed. Manual and digital intra-observer repeatability were excellent (ICC = 0.985 and ICC = 0.976, respectively). Manual measurements were on average 0.150 mm higher than digital measurements (SD = 0.407 mm; median = 0.145 mm; IQR = 0.29 mm), with 95% limits of agreement ranging from −0.647 mm to 0.947 mm. At the single-tooth level, 212 out of 480 measurements (44.2%) exceeded the predefined 0.3 mm threshold. First molars and selected premolars showed the highest discrepancy rates, whereas canines generally showed lower discrepancy rates. **Conclusions:** Manual and digital mesiodistal tooth-width measurements performed on digitized plaster models showed good agreement under the tested conditions. However, individual tooth-level differences exceeding the predefined 0.3 mm threshold were frequent, particularly for posterior teeth. Therefore, digital measurements obtained from digitized plaster models should be interpreted with caution in clinical situations requiring high precision at the level of individual tooth dimensions.

## 1. Introduction

Mesiodistal tooth width measurements constitute a fundamental component of orthodontic diagnosis, as they are used in tooth-size assessment, space analysis, and treatment planning. Accurate identification of individual tooth dimensions is particularly important in procedures requiring precise assessment of dental arch morphology and tooth-size relationships [1,2].

Traditionally, orthodontic measurements have been performed on plaster models obtained from alginate impressions. Studies indicate that traditional plaster models are still considered the reference standard in orthodontic diagnostics and treatment procedures [3,4]. They provide high precision and compatibility with articulators; however, their use is associated with several important limitations [4].

The conventional impression-taking process itself presents multiple challenges, including difficulties in selecting the appropriate tray size, the risk of material deformation, air bubbles, and marginal inaccuracies, as well as volumetric changes resulting from material shrinkage. In addition, this procedure is often associated with patient discomfort, a pronounced gag reflex, and a potential risk of aspiration [1,3,4,5].

Important limitations of plaster models also include the requirement for considerable storage space and their susceptibility to mechanical damage, such as fracture or chipping [3]. Additional disadvantages involve the time required for gypsum setting and the risk of porosity, which may negatively affect model quality [6]. Furthermore, plaster models do not allow easy sharing of documentation between clinicians and require physical transport, increasing the risk of damage and limiting their practicality in modern digital workflows [4,7].

The advent of intraoral scanners (IOS) and computer-aided design/computer-aided manufacturing (CAD/CAM) systems has provided solutions to many problems associated with traditional methods. Digital impressions eliminate the need for impression trays and materials, thereby reducing patient discomfort and minimizing the risk of material-related errors [1,5,6,7,8]. It should be noted that digital models may be obtained either by direct intraoral scanning or by digitization of plaster models, and these workflows are not equivalent, as clinical intraoral scanning is influenced by additional intraoral conditions not present during model digitization [7,9].

Studies indicate that digital models demonstrate high reliability and repeatability of measurements, with results generally comparable to those obtained from plaster models [3,6,10]. In both approaches, the identification of anatomical landmarks—rather than the measuring device or software—remains the primary factor influencing measurement precision [3,11].

Digital models offer several practical advantages, including improved model quality, increased patient comfort, simplified data storage, and the ability to share documentation electronically between clinicians [3,6]. Additionally, patients tend to prefer intraoral scanning due to greater comfort compared with conventional impression techniques [5].

While digital technology offers numerous benefits, it is not without limitations. The high cost of equipment represents a considerable barrier to adoption, particularly for smaller practices [3]. Scanning time may be prolonged, especially for inexperienced operators, and the effectiveness of the method largely depends on operator experience and adherence to appropriate scanning protocols [8,10]. Additionally, the presence of orthodontic appliances, saliva, and patient movement can affect scan quality [7,9].

Despite these limitations, measurements obtained from intraoral scanning, conventional plaster models, and digitized gypsum models have been shown to be generally comparable for most orthodontic parameters [6].

The performance of digital devices may vary depending on their type and application [11,12]. While numerous studies have compared digital and plaster models, there remains a need to evaluate the clinical relevance of inter-method differences, particularly at the level of individual tooth measurements. Previous evaluation methods based on physical measurements with calipers may be susceptible to human error [13,14]. In the present study, the 0.3 mm value for single-tooth measurements was adopted as a pragmatic threshold of potential clinical relevance, corresponding to the magnitude of inter-method differences reported in plaster–digital model comparisons [15]. However, only a limited number of studies have systematically evaluated whether differences between manual and digital measurements exceed predefined thresholds of potential clinical relevance at the individual-tooth level.

Furthermore, although digital models demonstrate high reliability and reproducibility, systematic inter-method differences have been reported [10,13,16]. The direction of inter-method differences varies across studies and depends on the digital workflow and measurement protocol. Shailendran et al. reported systematic underestimation of tooth widths in the ClinCheck Pro workflow [17], whereas studies comparing intraoral or digital models with plaster or conventional models have generally reported clinically acceptable agreement, despite small measurement differences [10,14,18]. The clinical implications of these differences, particularly for morphometric assessment of dental arches and individual tooth-size evaluation, require further investigation. The validity of repeated measurements with digital calipers in comparison with digital model measurements also requires further evaluation to ensure reliable morphometric analysis.

Therefore, the objective of the present study was to evaluate the agreement, repeatability, and inter-method differences between manual and digital measurements of mesiodistal tooth widths from the canine to the first molar on digitized plaster models, with particular emphasis on whether tooth-level discrepancies exceed a predefined threshold of potential clinical relevance.

## 2. Materials and Methods

This study was designed as an observational, ex vivo method-comparison agreement study evaluating the concordance between manual (digital caliper) and digital (intraoral scanner) mesiodistal tooth-width measurements performed on plaster dental models obtained from orthodontic patients.

The study was conducted in accordance with the Declaration of Helsinki and was approved by the Bioethics Committee of the Medical University of Silesia, Katowice (resolution no. BNW/NWN/0052/KB1/6/I/23, 4 April 2023). All plaster models were anonymized prior to analysis, and no identifiable patient data were accessed.

The material comprised diagnostic records of patients undergoing orthodontic evaluation at the Orthodontic Clinic of the Academic Centre of Dentistry and Specialized Medicine (ACSiMS Sp. z o.o.) in Zabrze, collected prior to initiation of orthodontic treatment. The documentation used in this study had been obtained previously as part of routine clinical diagnostics and was not collected specifically for research purposes.

Data were collected between May 2023 and May 2025. The sample included plaster models from 30 patients (19 female, 11 male; age range 11.5–17.0 years, mean age 14.2 ± 1.6 years) who met the eligibility criteria. Patients were selected consecutively from individuals presenting for orthodontic consultation.

The following inclusion criteria were applied:

- Permanent dentition from canine to first molar in both arches;
- Patients before initiation of orthodontic treatment;
- Good general health condition;
- Complete orthodontic diagnostic records (study models, panoramic radiograph, cephalogram);
- Fully erupted permanent teeth in analyzed segments.

The exclusion criteria were as follows:

- Craniofacial developmental anomalies;
- Ongoing orthodontic treatment;
- Dental anomalies affecting tooth number, size, or morphology, including hyperdontia, oligodontia, hypodontia, microdontia, macrodontia, and other developmental abnormalities, were excluded from the study;
- Impacted or reimpacted teeth;
- Teeth with extensive restorations that altered or obscured the natural anatomical landmarks;
- Ectopic teeth, or teeth outside the dental arch.

The demographic and clinical characteristics of the study sample are presented in Table 1.

No formal a priori sample size calculation was performed. The sample size was determined pragmatically based on the number of consecutively available plaster models that fulfilled the eligibility criteria during the study period. The final sample included 30 patients, which provided 480 paired single-tooth measurements. This sample size was considered sufficient for an exploratory method-comparison agreement analysis aimed at estimating inter-method differences and limits of agreement under controlled conditions. Nevertheless, the absence of an a priori sample size calculation is acknowledged as a limitation of the study.

For the purposes of this study, “early permanent dentition” was defined as fully erupted permanent teeth from the central incisor to the first permanent molar in all four quadrants, with no retained primary teeth and no teeth in active eruption in the analyzed segment.

Measurements were performed for each tooth from the canine to the first molar (teeth 13–16, 23–26, 33–36, and 43–46). The mesiodistal (MD) width of each tooth crown was measured using both manual and digital methods. Two calibrated examiners participated in the study. Manual measurements were performed independently by two different examiners. Digital measurements were performed twice by a single examiner, who was one of the two examiners performing the manual measurements. Prior to the main study, both examiners underwent a calibration session on five plaster models. Calibration was considered satisfactory when intra-examiner repeatability reached an ICC ≥ 0.90. To assess intra-observer repeatability, manual measurements (by each examiner) and digital measurements (by the single examiner) were repeated after a minimum 24 h interval, without reference to previous results. For inter-method comparison, the final manual value was calculated as the mean of the repeated manual measurements, whereas the final digital value was calculated as the mean of the two repeated digital measurements performed by the same examiner on the same digital model.

**Figure 1:**
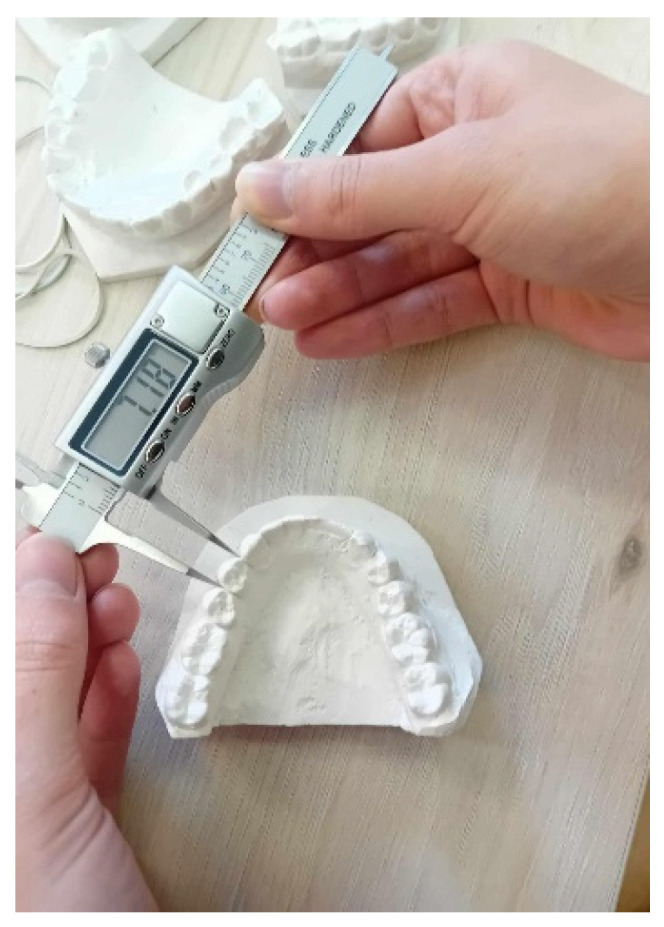
Manual mesiodistal tooth-width measurement performed on a plaster model using a digital caliper.

In the manual method, the plaster models were analyzed using a digital caliper (HSL 246-15) with a resolution of 0.01 mm and accuracy of ±0.02 mm up to 100 mm. The MD width was defined as the greatest distance between the anatomic mesial and distal contact points in a plane parallel to the occlusal surface (Figure 1).

Each plaster model was scanned once using a 3Shape TRIOS 4 intraoral scanner according to the manufacturer’s recommended protocol. Digital mesiodistal measurements were performed twice by the same examiner on the same digital model using 3Shape Unite platform version 1.7.33.2., with measurements recorded to the nearest 0.01 mm.

Therefore, intra-observer repeatability was assessed for both manual and digital measurements. However, because manual and digital measurements were not performed by the same examiner in a fully crossed design, the observed inter-method differences may still reflect both measurement-method differences and examiner-related variability.

The plaster models were scanned extraorally under stable indoor conditions, on a clean, dry surface, using diffuse overhead illumination. No scanning powder was used. The models were visually inspected before scanning to ensure the absence of visible defects, fractures, or surface contamination that could affect digital model acquisition.

The scanning sequence included the occlusal, buccal, and lingual/palatal surfaces of each model, with additional attention given to the interproximal areas of the teeth included in the study. Each model was scanned once; repeated scans were not performed. The resulting digital models were saved in standard tessellation language format (.STL) and imported into the 3Shape Unite platform, version 1.7.33.2, for linear measurements.

**Figure 2:**
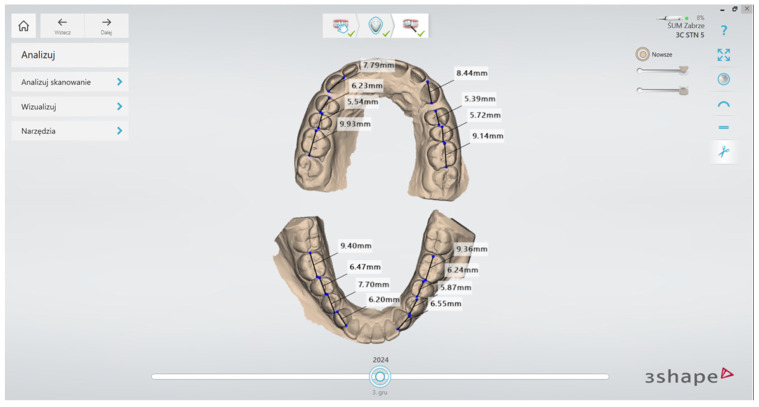
Digital mesiodistal tooth-width measurement performed on the digitized plaster model using 3Shape Unite platform version 1.7.33.2.

Digital mesiodistal measurements were performed twice for each analyzed tooth by the same examiner using the measurement tools available in the 3Shape Unite platform version 1.7.33.2, with measurements recorded to the nearest 0.01 mm (Figure 2). The same digital model obtained from the single scan was used for both repeated digital measurements.

A predefined threshold of 0.3 mm was adopted for single-tooth measurements as a pragmatic reference value corresponding to the magnitude of inter-method differences reported in previous comparisons of plaster and digital orthodontic models [15]. This value was not interpreted as a validated cut-off for clinical decision-making, but rather as a conservative marker of potentially relevant single-tooth measurement discrepancy.Statistical analysis was performed in Statistical analysis was performed in Python 3.12.3 using SciPy 1.17.1, NumPy 1.26.4, and Pingouin 0.5.5.

Intra-observer repeatability was assessed separately for manual and digital measurements by comparing the first and second measurements for each method, using the intraclass correlation coefficient (ICC) with a two-way mixed-effects model, single measures, and absolute agreement (ICC(3,1), according to Shrout and Fleiss and McGraw and Wong).

Inter-method agreement between the mean of the two manual measurements and the mean of the two digital measurements was evaluated using Bland–Altman analysis. For each tooth, the difference ΔMD was calculated as the mean manual measurement minus the digital measurement. Positive ΔMD values therefore indicated higher manual measurements, whereas negative values indicated higher digital measurements. Mean bias, standard deviation of the differences, median, interquartile range, and 95% limits of agreement (LoA = mean bias ± 1.96 × SD) were calculated overall and separately for each tooth type.

The number and percentage of measurements exceeding the predefined 0.3 mm threshold were calculated for each tooth. Because multiple teeth were measured within the same patient, tooth-level analyses were interpreted descriptively, and the clustered structure of the data was acknowledged as a limitation.

## 3. Results

A total of 480 teeth from 30 patients (16 teeth per patient) were included in the analysis. Intra-observer repeatability was assessed for both measurement methods. The intraclass correlation coefficient for repeated manual measurements (performed by two examiners) using a two-way mixed-effects model, single measures, and absolute agreement (ICC(3,1)) was 0.985 (95% CI: 0.981–0.988), indicating excellent intra-observer repeatability. Digital intra-observer repeatability (two repeated measurements performed by a single examiner on the same digital model) was also excellent, with an ICC(3,1) of 0.976 (95% CI: 0.970–0.981).

**Figure 3:**
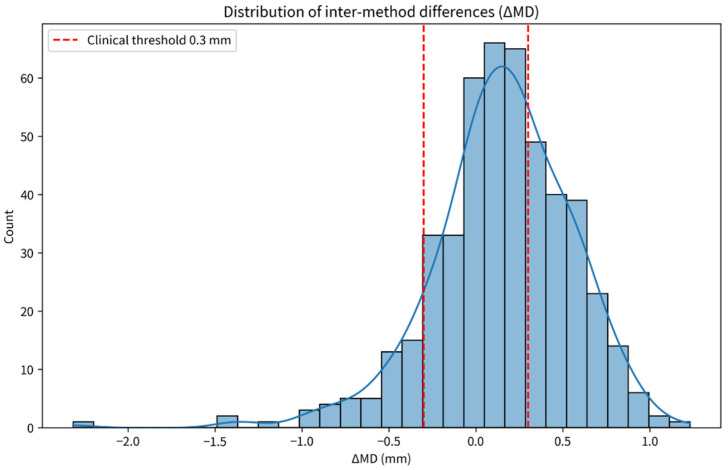
Bland–Altman plot showing agreement between mean manual and digital mesiodistal measurements (*n* = 480 teeth). The red dashed vertical lines indicate the predefined threshold of ±0.3 mm. ΔMD = mean manual − digital (mm).

Inter-method agreement between the mean manual and mean digital measurements was evaluated using Bland–Altman analysis. This revealed a systematic bias, with manual measurements being on average 0.150 mm higher than digital measurements (SD = 0.407 mm, median = 0.145 mm, IQR = 0.29 mm). The 95% limits of agreement ranged from −0.647 mm to 0.947 mm. The distribution of inter-method differences (ΔMD = mean manual − digital) is presented in Figure 3. In 212 out of 480 single-tooth measurements (44.2%), the absolute difference exceeded the predefined pragmatic reference value of 0.3 mm.

**Figure 4:**
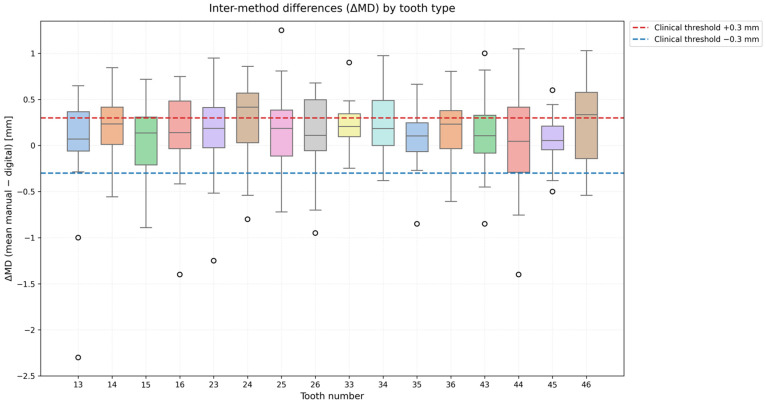
Box plots showing inter-method differences (ΔMD = mean manual − digital) according to individual tooth type. Boxes represent the interquartile range (IQR), horizontal lines indicate the median, whiskers represent the data range, and circles denote outliers. Different colors correspond to individual tooth types. Horizontal dashed red lines indicate the predefined clinical threshold of ±0.3 mm.

The detailed distribution of differences according to individual teeth was illustrated using box plots (Figure 4) and is further summarized in Table 2. Additional descriptive statistics for individual teeth corresponding to the distributions shown in Figure 3 and Figure 4 are provided in Supplementary Table S1. The analysis showed considerable variation in the percentage of differences exceeding the predefined 0.3 mm reference value depending on the tooth group. The largest discrepancies were observed for the first molars and selected premolars, including tooth 46 (22/30 measurements, 73.3%), tooth 24 (20/30, 66.7%), and teeth 25 and 44 (both 16/30, 53.3%). In contrast, canines and several premolars (particularly teeth 35, 45, 33, and 14) showed the lowest percentages of differences exceeding 0.3 mm (ranging from 23.3% to 33.3%). Tooth 13 was an exception among the canines, with 40% of measurements exceeding the threshold. When grouped by tooth type, first molars showed the highest percentage of measurements exceeding the predefined 0.3 mm threshold, whereas canines showed the lowest percentage. However, statistical comparison of ΔMD values between canines, premolars, and first molars did not demonstrate significant inter-group differences (Kruskal–Wallis test, p = 0.65; Supplementary Table S2).

Within this group, certain canines—particularly tooth 13—exhibited increased variability of measurement differences, demonstrated by wider interquartile ranges and isolated outliers, despite the low frequency of differences exceeding 0.3 mm. A subset of teeth, including teeth 15, 16, 26, 34, 36, and 43, displayed a heterogeneous, intermediate pattern. By contrast, tooth 44 demonstrated both a high proportion of differences exceeding the threshold and increased measurement variability.

## 4. Discussion

The ICC value obtained in this study (0.985 for repeated manual measurements) indicates excellent intra-observer reliability and is consistent with previous studies examining digital versus manual orthodontic measurements. Koretsi et al. reported substantial intra-observer reliability (ICC ≥ 0.7) for both manual and digital workflows, though they noted that 12.8% of manual measurements fell below this threshold, suggesting that digital methods may offer comparable consistency [13]. Similarly, Lippold et al. found excellent intra-, inter-, and repeated-measurement reliability when comparing CBCT-derived digital models with plaster casts, confirming that digital models may provide diagnostic performance comparable to traditional plaster models [19]. In a systematic review of 35 studies, Rossini et al. reported that digital models demonstrate comparable precision to traditional plaster casts and emphasized that the primary source of measurement error is related to landmark identification rather than the measuring system itself [11].

The observed systematic difference between manual and digital measurements in the present study is consistent with previously reported findings, although both the magnitude and direction of inter-method differences vary depending on the scanner system, measurement protocol, and digital workflow [11,13,16,17]. Similar inter-method variability has been reported across different orthodontic measurement techniques, supporting the concept that measurement differences are influenced not only by the method itself, but also by operator-dependent factors and landmark identification [3,10]. Similarly, Jiménez-Gayosso et al. found that manual and digital measurements of dental arches showed overall similarities and strong correlations, although statistically significant differences were observed for selected transverse, vertical, and arch-perimeter measurements [20]. It should be noted that several cited studies involved direct intraoral scanning or CBCT-derived models; therefore, their results are not directly comparable to the plaster-model digitization workflow applied in the present study [6,9,19].

At the single-tooth level, 44.2% of measurements exceeded the predefined 0.3 mm reference value. The highest proportions of differences were observed for first molars and selected premolars, whereas canines demonstrated the lowest level of inter-method discrepancy. A similar anterior–posterior pattern has been reported in scanner-performance studies, with intraoral scanner accuracy tending to decrease from anterior to posterior teeth [21,22]. This pattern may be explained by the greater anatomical complexity of posterior crowns, increased difficulty in reproducing proximal and occlusal morphology, and patient-related intraoral factors such as tooth type, wetness, interdental spaces, and surface characteristics [21,22,23]. In addition, operator experience, scanner type, scan size, and the length of the scanned distance may influence scan accuracy and contribute to local discrepancies in digital models [24,25]. In vivo scanner studies also indicate that, despite generally acceptable overall trueness and precision, local imprecision may occur in specific regions of the arch [26]. Additionally, dental crowding itself may reduce the trueness and precision of intraoral scans, particularly in severe crowding, where narrow interproximal spaces make accurate data capture more difficult [27].

Previous studies have shown that digital and manual measurements may differ systematically; however, as neither method represents a true reference standard, such differences should be interpreted as relative inter-method bias rather than absolute underestimation or overestimation [10,16]. In the present study, digital measurements were lower than manual measurements under the conditions of this study, with a mean difference of approximately 0.15–0.20 mm. These findings are consistent with prior observations, although the reported direction of differences varies across studies depending on the measurement workflow and analytical approach [10,16]. Similarly, Kardach et al. reported small but statistically significant differences between plaster and digital model measurements for selected teeth, with mean tooth-width differences reaching approximately 0.30 mm for some teeth, while concluding that these differences were clinically acceptable overall [15].

From a clinical perspective, the findings of this study suggest that digital measurements obtained from digitized plaster models may be acceptable for routine tooth-width assessment under the tested conditions. However, procedures requiring higher precision at the level of individual teeth may require careful interpretation of digital measurements, particularly in posterior regions where discrepancies were more frequent.

Several limitations should be acknowledged. The sample size was relatively small and determined pragmatically, without a formal a priori sample size calculation, which may limit the precision and generalizability of the agreement estimates. No true reference standard was available; therefore, the results reflect agreement between methods and relative inter-method bias rather than absolute measurement accuracy. Although both manual and digital measurements were repeated and showed excellent intra-observer repeatability, the study did not use a fully crossed design in which each examiner measured both modalities. Therefore, the observed inter-method differences may still partly reflect examiner-related landmarking variability.

The clustered structure of the data, with multiple teeth nested within the same patient, may partially violate the assumption of independent observations. Therefore, tooth-level findings should be interpreted descriptively, and future studies should consider mixed-effects models or generalized estimating equations. Furthermore, only one scanner system was evaluated, and measurements were performed on digitized plaster models rather than under direct intraoral conditions. Detailed clinical characteristics such as arch form, degree of tooth rotation, posterior crowding severity, and alignment complexity were not systematically analyzed; therefore, the generalizability of the findings to patients with severe crowding or pronounced posterior rotations may be limited.

Future studies should include repeated digital measurements and a fully crossed design in which each examiner performs both measurement methods. Additionally, studies conducted under direct intraoral conditions and involving multiple scanner systems would provide further insight into the clinical applicability of digital measurements.

## 5. Conclusions

Manual caliper measurements demonstrated excellent intra-observer repeatability, and manual and digital mesiodistal tooth-width measurements performed on digitized plaster models showed good agreement under the tested conditions.

Digital measurements obtained using the evaluated scanner tended to yield lower mesiodistal tooth-width values than manual measurements. Although the mean inter-method difference was small, differences exceeding the predefined 0.3 mm threshold were frequent at the individual-tooth level, particularly for first molars and selected premolars.

These findings suggest that digital measurements on digitized plaster models may be acceptable for general tooth-width assessment under controlled conditions. However, caution is warranted when interpreting individual posterior tooth dimensions in clinical situations requiring high measurement precision. The conclusions are limited to the tested scanner system, digitized plaster models, selected teeth from canine to first molar, and the measurement protocol used in this study.

## 6. Clinical Relevance

Digital measurements obtained from digitized plaster models may be acceptable for routine assessment of mesiodistal tooth widths under controlled conditions. However, individual tooth-level discrepancies, particularly in posterior teeth, should be interpreted with caution. When precise assessment of posterior tooth dimensions is critical for diagnosis or treatment planning, verification with direct clinical or model-based assessment may be considered.

## Figures and Tables

**Table 1:** Demographic and clinical characteristics of the study sample (*n* = 30).

Variable	Value
Sex, *n* (%)	
Female	19 (63.3)
Male	11 (36.7)
Age (years), mean ± SD (range)	14.2 ± 1.6 (11.5–17.0)
Number of teeth analyzed per patient	16
Total number of teeth analyzed	480

**Table 2:** Descriptive statistics of inter-method differences (ΔMD = mean manual − digital) at the individual tooth level (*n* = 30 per tooth).

Tooth	Mean ΔMD (mm)	Median ΔMD (mm)	SD (mm)	95% LoA (mm)	% >0.3 mm
13	0.000	0.070	0.582	−1.141 to 1.141	12/30 (40.0%)
14	0.220	0.235	0.301	−0.369 to 0.810	10/30 (33.3%)
15	0.065	0.138	0.402	−0.723 to 0.852	14/30 (46.7%)
16	0.153	0.140	0.444	−0.717 to 1.022	13/30 (43.3%)
23	0.189	0.188	0.455	−0.703 to 1.081	13/30 (43.3%)
24	0.280	0.418	0.422	−0.547 to 1.107	20/30 (66.7%)
25	0.167	0.188	0.429	−0.673 to 1.008	16/30 (53.3%)
26	0.125	0.110	0.400	−0.659 to 0.910	13/30 (43.3%)
33	0.232	0.208	0.258	−0.273 to 0.737	9/30 (30.0%)
34	0.239	0.185	0.344	−0.436 to 0.913	13/30 (43.3%)
35	0.100	0.105	0.317	−0.520 to 0.720	7/30 (23.3%)
36	0.174	0.232	0.339	−0.491 to 0.840	15/30 (50.0%)
43	0.138	0.108	0.404	−0.654 to 0.930	11/30 (36.7%)
44	0.017	0.048	0.517	−0.997 to 1.031	16/30 (53.3%)
45	0.055	0.055	0.265	−0.464 to 0.574	8/30 (26.7%)
46	0.249	0.335	0.450	−0.633 to 1.130	22/30 (73.3%)

## Data Availability

The data presented in this study are available on request from the corresponding author.

## Supplementary Materials

The following supporting information can be downloaded at: https://www.mdpi.com/article/10.3390/jcm15155911/s1, Table S1. Descriptive statistics of mesiodistal measurement differences (ΔMD) by tooth; Table S2. Mesiodistal measurement differences (ΔMD) grouped by tooth type.
