# Supplementary material for: Agreement Between Manual and Digital Mesiodistal Tooth Measurements on Digitized Plaster Models: A Tooth-Level Analysis
Source: J Clin Med. 2026 Jul 29;15(15):5911. doi: 10.3390/jcm15155911 (PMC13466334; doi:10.3390/jcm15155911)
Supplement: Supplementary file 1 [file jcm-15-05911-s001.zip › jcm-4384405-supplementary.pdf]

Supplementary

Table S1. Descriptive statistics of mesiodistal measurement differences (ΔMD) by tooth

(ΔMD = Manual − Digital; positive values indicate higher values obtained with the manual method than with the digital method)

Tooth	Mean ΔMD (mm)	SD (mm)	Median ΔMD (mm)	% of measurements |ΔMD| > 0.3 mm
13	0.00	0.58	0.14	40.0
14	0.22	0.30	0.21	33.3
15	0.06	0.40	0.05	46.7
16	0.15	0.44	0.11	43.3
23	0.19	0.46	0.17	43.3
24	0.28	0.42	0.30	64.5
25	0.17	0.43	0.19	51.6
26	0.13	0.40	0.12	43.3
33	0.23	0.26	0.24	30.0
34	0.24	0.34	0.22	43.3
35	0.10	0.32	0.09	23.3
36	0.17	0.34	0.15	50.0
43	0.14	0.40	0.12	36.7

44	0.02	0.52	0.03	51.6
45	0.05	0.26	0.04	26.7
46	0.25	0.45	0.27	70.0

Table S2. Mesiodistal measurement differences (ΔMD) grouped by tooth type

(ΔMD = Manual − Digital; positive values indicate higher values obtained with the manual method than with the digital method)

Tooth group	Teeth included	Mean ΔMD (mm)	SD (mm)	Median ΔMD (mm)	% of measurements |ΔMD| > 0.3 mm
Canines	13, 23, 33, 43	0.14	0.48	0.14	37.5
Premolars	14, 15, 24, 25, 34, 35, 44, 45	0.14	0.36	0.15	43.3
First molars	16, 26, 36, 46	0.18	0.39	0.19	51.7

Descriptive analysis of mesiodistal measurement differences grouped by tooth type (Table S2) revealed a clear clinical pattern. Canines demonstrated the smallest mean ΔMD values and the lowest frequency of clinically significant discrepancies, whereas first molars showed larger mean deviations, higher variability, and the highest proportion of measurements exceeding the 0.3 mm predefined 0.3 mm

threshold of potential clinical relevance. Premolars exhibited intermediate values between these two groups.

However, statistical comparison of ΔMD values between canines, premolars, and first molars did not demonstrate a significant difference among tooth groups (Kruskal–Wallis test, $p = 0.65$). This indicates that, despite observable differences in the magnitude and frequency of clinically relevant discrepancies, the variability within each tooth category was substantial and outweighed between-group differences. From a clinical perspective, these findings suggest that tooth-type-dependent trends are more relevant for identifying patterns of measurement behavior than for defining statistically distinct categories. In particular, the higher frequency of clinically significant discrepancies observed for first molars may have practical implications when high single-tooth precision is required, even in the absence of statistically significant inter-group differences.
